# Supplementary material for: Global supply chains amplify economic costs of future extreme heat risk
Source: Nature. 2024 Mar 13;627(8005):797–804. doi: 10.1038/s41586-024-07147-z (PMC10972753; doi:10.1038/s41586-024-07147-z)
Supplement: Supplementary file 1 — Supplementary sections 1–7, including Figs. 1–13, Tables 1–7 and references. [file 41586_2024_7147_MOESM1_ESM.docx]

Supplementary Materials for

**Global supply chains amplify economic costs of future extreme heat risk**

Yida Sun et al.

Corresponding authors: Dabo Guan [guandabo@tsinghua.edu.cn](mailto:guandabo@tsinghua.edu.cn);

**The PDF file includes:**

Supplementary Sections 1 to 7

Supplementary Figures 1 to 13

Supplementary Tables 1 to 7

References

# Robustness tests and validation

## Uncertainty and validation of health loss

The uncertainty of heatwave related mortality is propagated through the uncertainty of relative risk (RR) used in equation 4 (See Extended Data Table 1). The uncertainty of RR determined by the meta-analysis conducted by Guo et al.^1^ on heatwave related mortality data from 400 communities across 18 counties/regions with different climates, where 12 different RR groups are calculated based on various heatwave definitions (See Table 1 in Guo et al.). In the main text we adopted the less restrictive heatwave definitions of above 95% mean temperature with ≥2 days. To further test the robustness and uncertainty associated with heatwave definition, we conducted additional experiments with the most restrictive heatwave definition available in Guo et al., which is above 97.5% mean temperature with ≥4 days (See Extended Data Table 2 for related RR).

To validate our estimation, we obtained 42 heatwave records with reported heat-related mortality greater than 100 between 2000-2022 from EM-DAT^2^ (The international disaster Database) and national statistics including heatwave mortality monitoring report 2016-2020 from Public Health England (<https://www.gov.uk/government/publications/phe-heatwave-mortality-monitoring>) etc.. Most of those records are from Europe (35 out of 42) with 19 from Western Europe, 8 from Southern Europe, 4 from Northern Europe, and 4 from Eastern Europe. The rest of the records include 4 from Southern Asia, 1 from Northern America, and 2 from Oceania.

We calculated the heatwave mortality for each corresponding event based on the grided daily observational surface temperature at 0.25° resolution obtained from ERA5^3^ and population data from GPWv4^4^ for both heatwave definitions. Detailed comparisons for those 42 events can be found in the supplementary file (Supplementary Table6. compiled heat stress cost information for validation). Supplementary Table 1 presents the comparison between model results and reported mortality for different regions. Relatively good matches are found for North America, Northern Europe, and Oceania. Underestimations are found for Western (~30%), Southern (~65%) and Eastern Europe (~55%). Overestimations are found for Southern Asia. In total, we found our estimation caches about 75% of the reported death for all 42 events. We also noticed that the 95% heatwave definition results in less bias than the 97.5% heatwave definition compared to the reported values.

We also assess our model performance for some of the most impactful and well-known heatwave events between 2000 and 2022 as presented in Supplementary Figure 1. Reasonable model performance is found for half of all events, where we find good perdition for Europe 2022 and Western Europe 2020 based on the 95% heatwave definition, while the reported death from the Western Europe 2006 and Northern America 2021 events are within the uncertainty range of model results based on the 97.5% heatwave definition. The death tolls are underestimated for the Europe 2003, and Russia 2010 heatwave events which are also the top 2 most deadly heatwave events in the study period. Overestimations are found for events including India-Pakistan 2015 and West Europe 2019 heatwave events. In general, it seems our model tends to underestimate heatwave events with extremely high casualties while more likely to overestimate low casualty events.

Due to the huge uncertainty associated with heatwave mortality, it is always challenging to validate the accuracy of model estimation, and we have not identified yet another study in the field try to compare their modeling results with reported death values. Our simplified four-climate-zone-based estimation is likely to neglect some important sub-regional characters and should be interpreted with caution, as there are many additional factors not included in this study such as air condition accessibility^5^, age^6^, humidity^7^ etc.

Supplementary Table 1 Comparison between reported heatwave mortality and modeled results between 2000 and 2022 in different regions.

| Regions | Even Count | Reported Death | 95% 2 Day Death | 97.5% 4Day Death |
| --- | --- | --- | --- | --- |
| Oceania | 2 | 486 | 659  (510 - 809) | 211  (165-257) |
| Northern America | 1 | 815 | 1178  (899 - 1460) | 648  (532 - 766) |
| Southern Asia | 4 | 4687 | 40409  (29349 - 51748) | 18723  (12160 - 25384) |
| Eastern Europe | 4 | 56930 | 24263  (18599-29992) | 21900  (17789 - 26070) |
| Northern Europe | 4 | 6888 | 9112  (6939 - 11311) | 5940  (4880 - 7012) |
| Southern Europe | 8 | 45132 | 14487  (11256 - 17751) | 12148  (9451 - 14907) |
| Western Europe | 19 | 52150 | 34655  (26492 - 42915) | 22809  (18582 - 27094) |
| Total | 42 | 167088 | 124762  (94042 - 155987) | 82378  (63558 - 101490) |


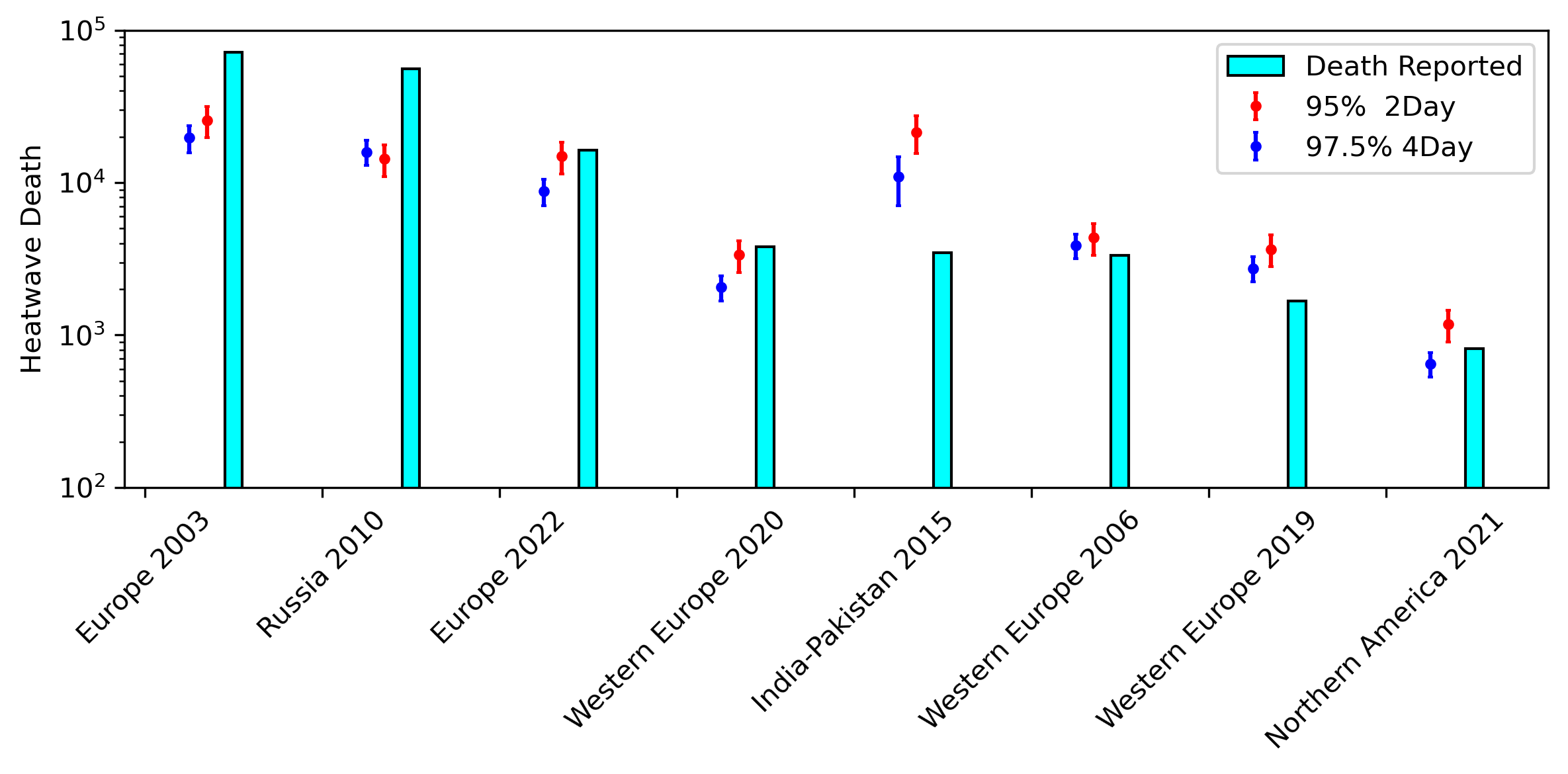


**Supplementary Figure 1 Comparison between reported death and modeling results for major heatwave events between 2000-2022**

The uncertainty in VSL is also evaluated through an additional sensitivity test, which assumes equally valuation of life across the globe. For such a test, an averaged VSL is calculated by multiplying each country's income-based VSL by its population, summing the results, and dividing by the world's total population. Upon compiling and analyzing the data, we determined a global average VSL of 2.89 million USD in year 2020. We use the globally equal VSL calculation above as a reference.

Under a future global average VSL, the economic inequality of global health losses would become more pronounced due to the higher value of health losses in most LDCs compared to the value of health losses in most developed countries. Our findings indicate that global economic losses are 5-20% lower under a global ‘equal VSL’ compared to a global ‘heterogeneous VSL’. (Supplementary Figure 2). This is because the global average VSL is significantly lower than the ‘heterogeneous VSL’ in heatwave vulnerable countries such as Russia, the USA and France, which have large populations and a high number of excess deaths due to heatwaves annually. Economic losses in these three countries would be reduced by 2 to 8 times under ‘equal VSL’. Conversely, economic losses in heatwave-vulnerable countries like India, Bangladesh and Ethiopia would be 2 to 7 times higher under ‘equal VSL’, as the ‘heterogeneous VSL’ is 1.2 million USD for India and 0.4 million USD for Ethiopia.

The distribution of economic losses as a percentage of national GDP for each country under the two VSL approaches is illustrated in Supplementary Figure **3** (patterns under SSP119 and SSP245 scenarios are comparable). The ‘equal VSL’ assumption reveals that the inequality of health losses from climate change is likely to be greater in a future scenario where developing countries value human capital more than they currently do (approaching the global average). In in many Latin American and African countries, for instance, economic losses amount to more than 10% of their GDP by midcentury. By 2060, economic losses in Tajikistan would represent 25.2% of GDP, while in Malawi, they would account for 65.9% of GDP. Consequently, without a coordinated global effort to reduce emissions, deaths due to warming and heatwaves in developing countries are likely to incur an increasingly high economic cost. We have adopted the heterogeneous VSL as benchmark VSL in the main text and used the global average VSL results as a reference for the supporting material.


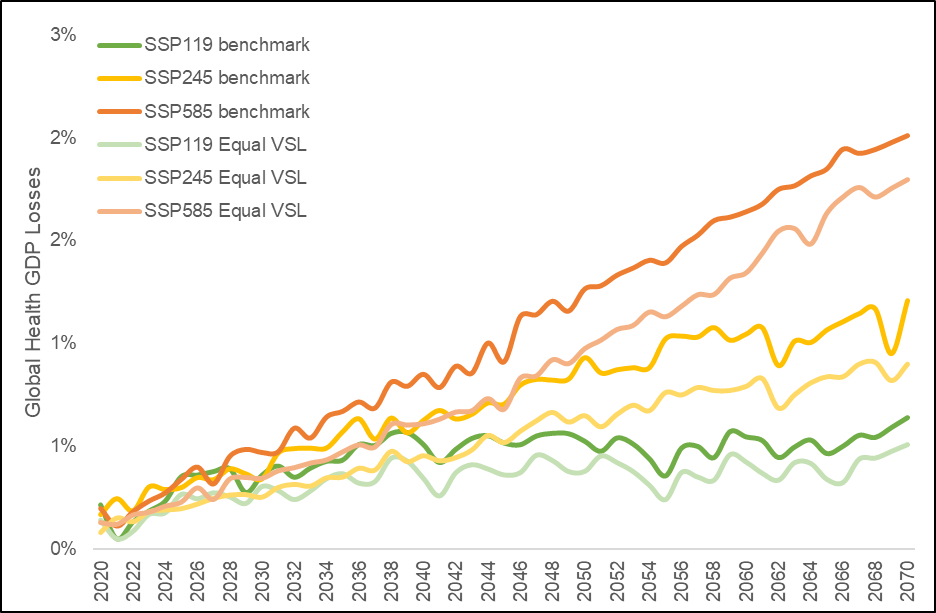


**Supplementary Figure 2 Evolutionary trends of global health losses from heat wave fatalities using two VSL settings.** The three dark curves represent the results of the calculations using the classical differentiated VSL for the three scenarios SSP119 (green), SSP245 (yellow) and SSP585 (orange) respectively. The lighter curves represent the results of the calculations using the global average VSL.


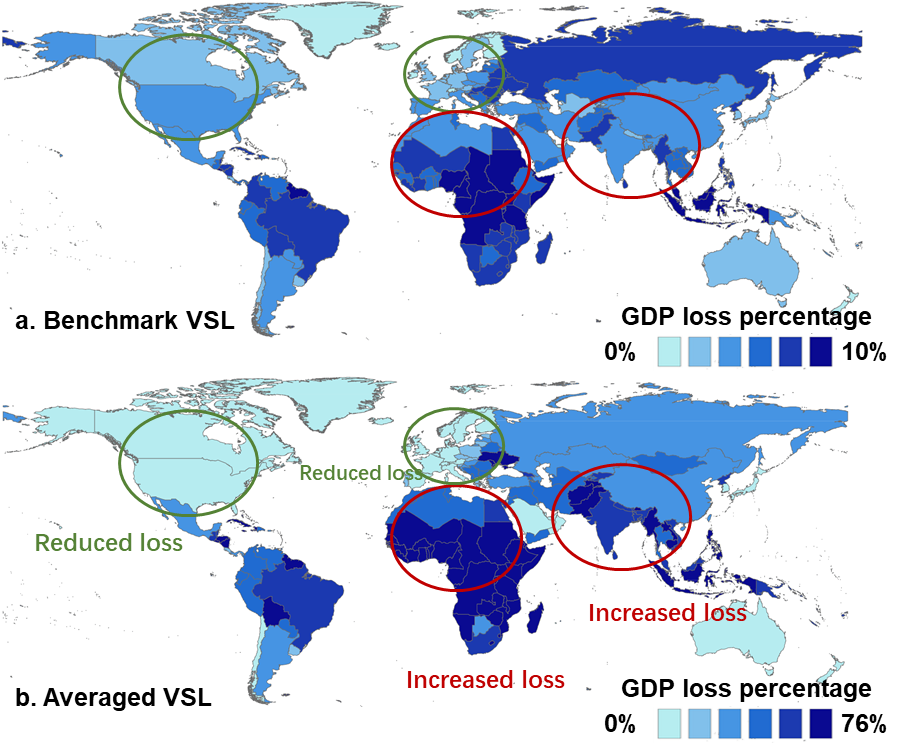


**Supplementary Figure 3 Spatial patterns of global health losses from heat wave mortalities using two VSL settings under SSP585 scenario, year 2060.**

Overall, South Central Africa, Southeast Asia, Eastern Europe and Central America have relatively high losses under multiple robustness tests.

1. **Eastern European countries’ heatwave sensitivity:** For example, Russia is susceptible to heat waves because of low adaptability to extreme heat. Its average daily mortality rate between 2009-2019 stands at 1.46%, making it one of the highest globally. Only Lesotho (1.66%), Bulgaria (1.50%), and Ukraine (1.48%) surpass it. In addition, some Eastern European countries, such as Ukraine, have populations concentrated in moderately cold climatic zones, where the relative risk values for heat waves are highest. This statistic, based on equation (4) in the SI, indicates Eastern Europe's pronounced vulnerability to heatwave risks. For example, the 2010 summer heatwave in the Northern Hemisphere resulted in approximately 55,000 heatwave-related deaths in Russia^8^, underscoring the potential for substantial heatwave-related fatalities in the region.
2. **Heatwave Duration in Southern Africa, Southeast Asia and Central America:** The increased losses in southern Africa, especially in Angola, the Democratic Republic of the Congo and Indonesia, are primarily due to longer heatwave durations attributable to global warming (as shown in Extended Data Fig. 2). South-central Africa's rise in heatwave days is among the most substantial globally. This trend aligns with another study based on CMIP5 projections^9^.

Nonetheless, our estimates of health loss have import limitations as numerous studies have confirmed that older people (65+ years) are more sensitive to heatwaves in terms of health loss ^10^, potentially experiencing more than twice the mortality rate of middle-aged people based on empirical studies in China, Australia and other regions ^11,12^. Because we used an all-age dataset, our health loss estimates are conservative, given the increasing global aging trend.

## Uncertainty and validation of labor productivity loss

For the uncertainty analysis of labour productivity losses, we used the Hothaps labor loss function as a comparison. Where the set of parameters α1 and α2 were equal to (34.64, 22.72) for low, (32.93, 17.81) for moderate and (30.94, 16.64) for high workload, respectively^13^.

In terms of the assessment of the absolute labour loss rate, the Hothaps logistic function has lower results than the ERF function due to the assumption that at least 10% of labour time is retained, i.e., working is possible for 6 minutes within each hour even under extreme heat. The Hothaps logistic function results in a slightly lower total loss assessment of around 5%-12% compared to the ERF function. However, this study assesses the additional amount of damage caused by climate change. The difference in calculation of the function itself is offset when subtracted from the base period. So the global labour losses calculated using the two functions are therefore very close (within 5% of each other) for the uncertainty analysis. Even when looking at the subdivision of losses per country per sector, the two functions give similar results (Supplementary Figure **5**) in measuring climate change labour losses and largely match in terms of relative loss size ranking. The comparison between two functions is shown in Supplementary Figure 4 and Supplementary Figure 5, using the construction sector as an example.


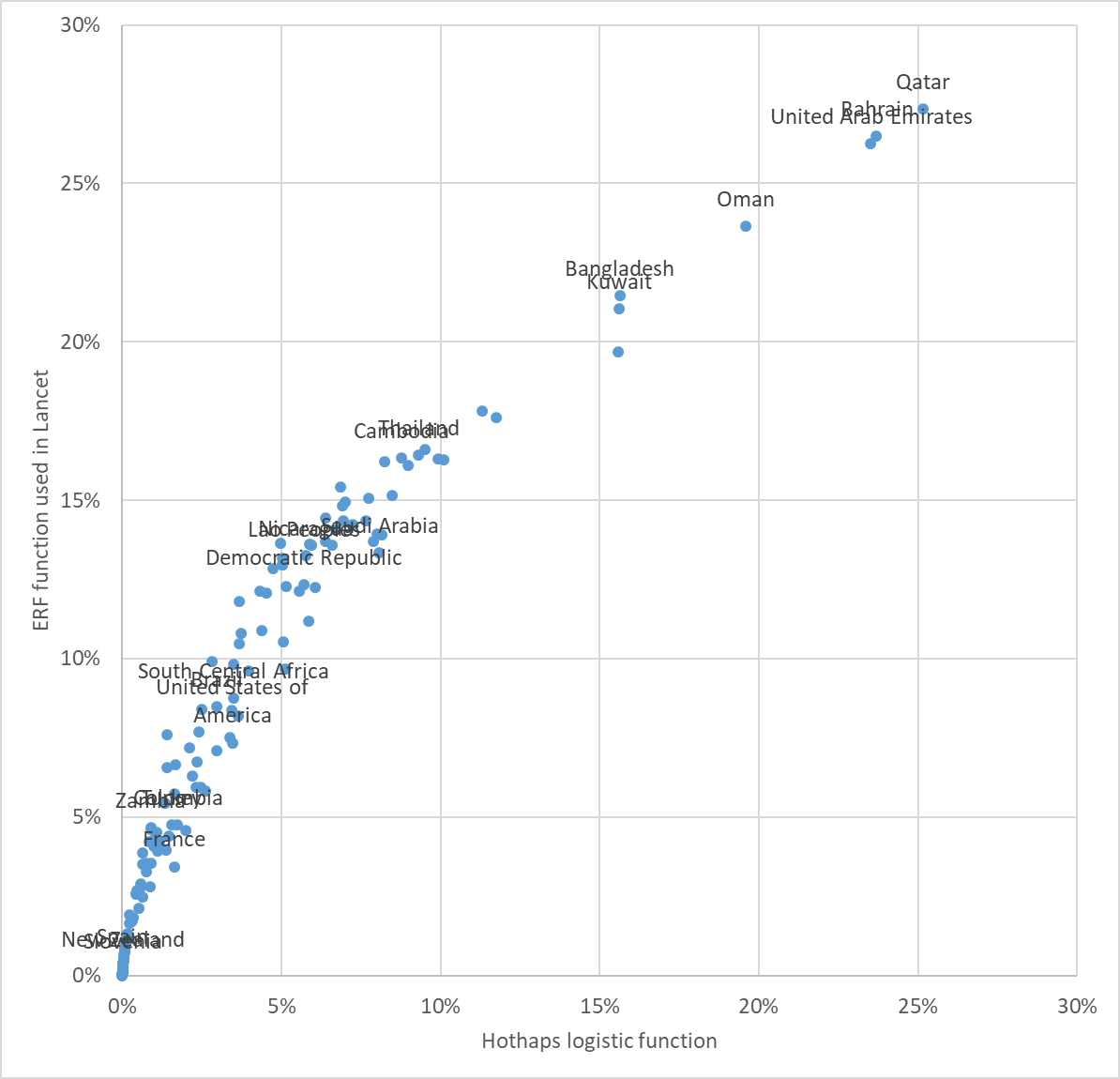


**Supplementary Figure 4 Labour productivity loss proportion in construction sectors across countries assessed by two functions in 2015 based on ERF function (y-axis) and Hothaps logistic function (x-axis).**


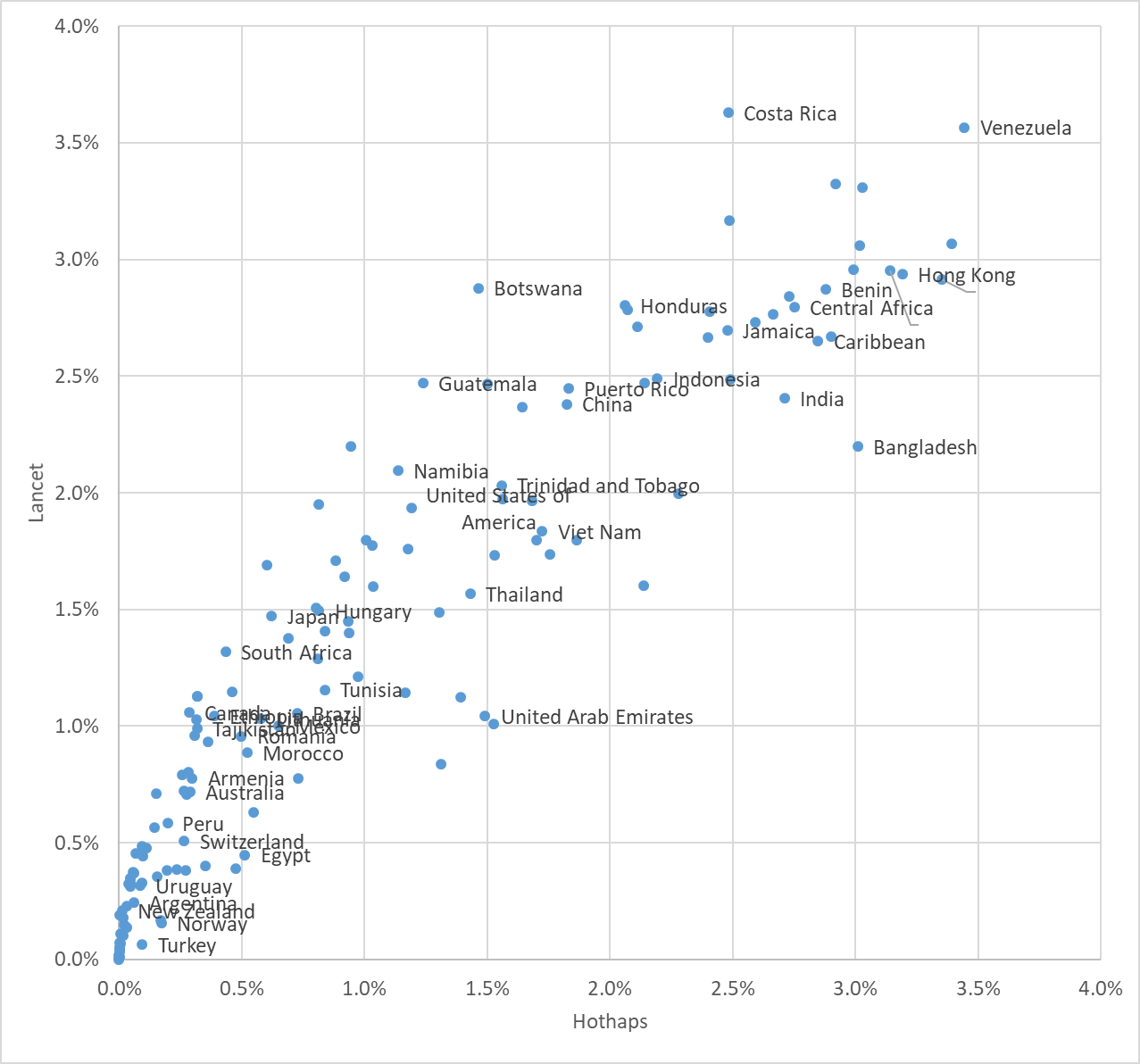


**Supplementary Figure 5 Climate change induced labour productivity loss proportion in construction sectors in 2060 across countries under the SSP119 scenario based on ERF function (y-axis) and Hothaps logistic function (x-axis).**

Our assessment of the economic damage caused by reduced labour productivity matches well with existing literature, and with a 10% to 30% difference with some institutional reports.

For example, the report of Rockefeller Foundation Resilience Center found that worker productivity losses totaled approximately $100 billion in the United States in 2020. In comparison, we estimate that the productivity losses was $72.1 billion. Economic research company Prognos’ report (cited by Germany's economy and environment ministries) shows that nine billion euros in damages were caused by workers' lower productivity in industry and commerce sectors due to heat waves in 2018 and 2019. In comparison, we estimate that the productivity losses was 10.7 billion euros.

In comparison with existing literature, since studies related to labour productivity losses have used statistically based exposure response functions, the assessment results are relatively close. The difference between studies often lies in adaptive technology assumptions, such as the prevalence of air conditioning and mechanisation. Our calculations of the global GDP loss due to labour productivity decline (0.4%, 0.6%, 0.8% in 2040, 2050, 2060 respectively, SSP585) are consistent with research by Anton Orlov et al^14^ (0.3%, 0.5%, 0.7% in 2040, 2050, 2060 respectively, RCP8.5, adjusted to 2015 benchmark). While in reports of the United Nations’ International Labor Organization (air conditioning and mechanisation not considered), more than 0.8 percent of working hours worldwide will be lost every year by 2030 compared to 1995. Although mechanisation was not considered in our study, the comparisons in Orlov’s study revealed that it would only make a difference of approximately 3% to 5% over the time frame of this study.

Despite partial validation from historical data and the literature, the methodology for calculating labor losses still has limitation in future estimations. For example, we do not consider workers’ differential physiological resilience to high temperatures in individual countries or the different parameters for the more subdivided types of work within specific industries. In addition, the simplified WBGT model only quantify the effects of changes in temperature and humidity due to climate change. Other meteorological variables, such as strong radiation or low wind speed, can exacerbate heat stress, although it primarily depends on temperature and humidity ^15,16^. The simplified formula assumes moderately high levels of heat radiation in light wind conditions, disregarding long-term trends of changes in solar radiation and wind speed.

## Uncertainty and validation of indirect loss

Uncertainty about indirect losses arising from (1) production side, which includes the base period production and trade structure, inventories, excess production capacity and the elasticity of trade substitution of products across countries; and (2) demand side, which includes the ability of the final demand account to adjust when there is a shortfall in demand or supply. While the ARIO model is widely used and work well for a single-country/single-region analyses, the substitutability of products in a multi-country scenario requires further discussion to verify robustness. To address the uncertainty in the structure of production and trade, we have used different years and versions of the input-output database for comparison tests (see Extended Data Fig. 4). For parameters such as the maximum stock ratio and excess production capacity, we repeated the experiment several times within the range of possible values from previous studies. For trade substitutability, upper and lower bounds of perfect substitution and non-substitution (traditional static IO model) were used.

Estimates are displayed as 10-year averages for the year 2060, using the GTAP 2011 (a), GTAP 2014 (b) and EMERGING 2019 (c) databases separately. The colours of the bars represent GDP per capita from low to high. (d), indirect losses under different benchmark trade structures in each region. The horizontal axis measures the indirect losses as a percentage of GDP using the GTAP2014 trade structure and the vertical axis measures the indirect losses as a percentage of GDP using the GTAP2011 trade structure.


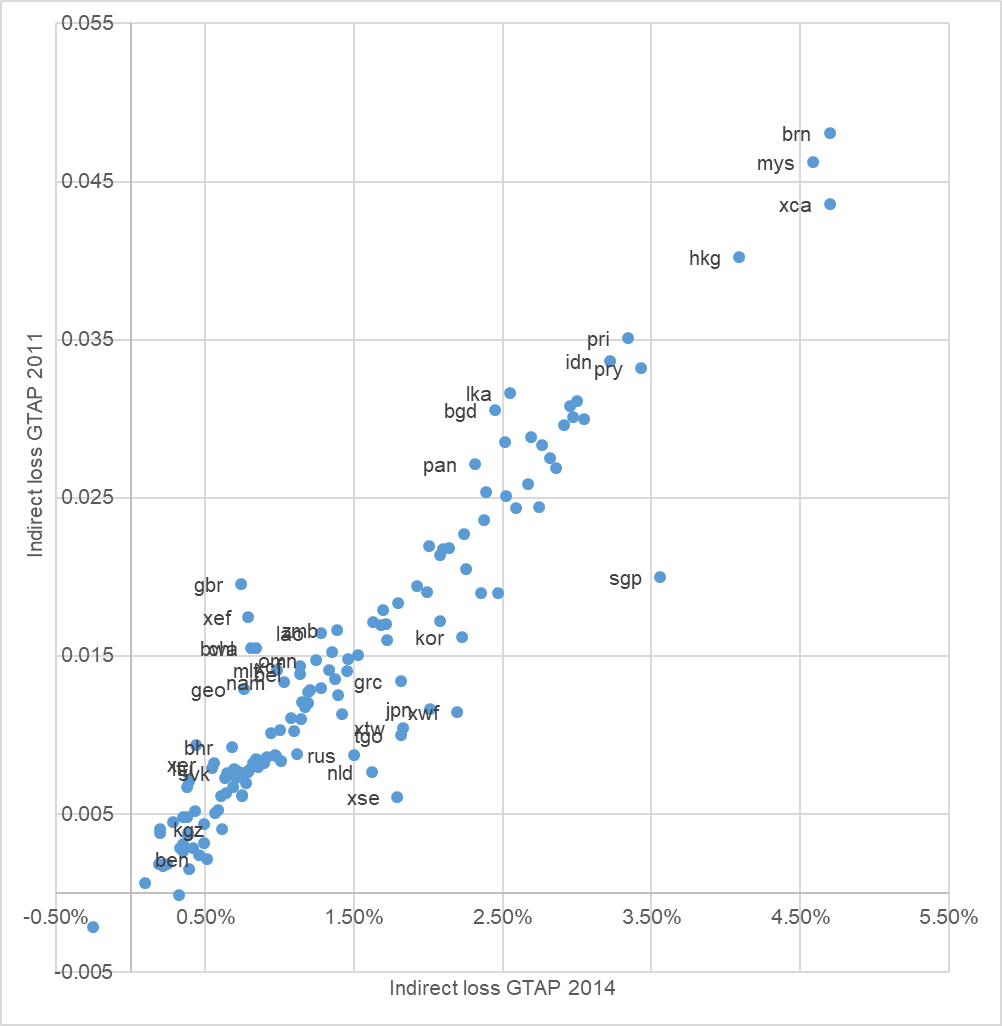


**Supplementary Figure 6** Comparison of the impact of using different trade structures on the assessment of indirect losses using GTAP2011 and GTAP2014. The horizontal axis is the indirect losses as a percentage of GDP using the GTAP2014 trade structure and the vertical axis is the result of using the GTAP2011 trade structure.

Globally, the total amount of indirect losses remains robust to changes in the structure of the data over the base period. The results of the global scale loss assessment differ by less than 5% in 2060. The vast majority of countries are distributed around the y=x line, which implies a consistent assessment across different trade structures.

Regionally, for a small number of countries, indirect loss assessments can show larger differences. By comparison, we find that when using GTAP 2014 data for the base period, indirect economic losses in East and Southeast Asian countries, such as Singapore, Korea, Japan, are amplified (see Extended Data Fig. 4 and Supplementary Figure6). This can be explained by the fact that, in GTAP2014, those countries have closer economic ties with climate-sensitive markets, including Malaysia, China, India and Vietnam. For instance, trade between Singapore and emerging economies like China and Vietnam had increased substantially from 2010 to 2014. According to the Singapore Department of Statistics (https://www.singstat.gov.sg/) and the United Nations Commodity Trade Statistics Database (https://comtrade.un.org), China became the largest trading partner of Singapore in 2014, up from 4th place in 2011, whereas Vietnam rose to the 13th largest partner in 2014, from the 20th place in 2011. Conversely, Singapore's total trade share with the EU and the USA decreased slightly over the same period. Similarly, Japan, Korea and Myanmar developed closer trade relationships with emerging markets such as China, India, and Vietnam.

The assessment of the different trade structures brings important insights, into the need for both developed and emerging developing countries, which will be increasingly involved in international trade in the future, to carefully consider the supply chain risks posed by climate.

We elaborate in more detail the uncertainty parameter intervals for the three main modules and do a Monte Carlo analysis, including simulation of economic loss dynamic for 10000 periods. We have also summarized the results of previous assessments of different models based on CMIP5 data and similar RCP scenarios for comparison. The results are shown in Extended Data Fig. 5.

Supplementary Table 2 The set of parameters for the uncertainty test under the three loss modules

| Loss modules | Parameters | Range |
| --- | --- | --- |
| Health Loss | Relative Risks | See ED Table 2 |
|  | Heatwave definition | {95%, 97.5%} ,{2d, 4d}, {dynamic, static} |
|  | Climate models | See ED Table 1 |
|  | VSL | {viscusi 2020, averaged} |
| Labor productivity loss | Labor loss function | {Hothaps, ERF} |
|  | Maximum overproductivity | (0,25%] |
|  | Overproduction Step | (0,5%] |
|  | Time scope | {HWDs, hot season} |
| Indirect loss | Stock magnitude | (0,1.5] |
|  | Trade substitutability | {0,5%,10%,20%,100%,CGE} |
|  | Maximum percentage decrease in final demand under supply shortage | [0,10%] |
|  | Maximum percentage increase in final demand under insufficient demand | [0,10%] |
|  | Trade Structure | {GTAP2014, GTAP2011, EMERGING 2019} |

Supplementary Figure **7** a-d is the high adaptation scenario, with lowest economic losses. The scenario parameters for the high adaptation scenario losses are set as follows. The country provides producers with a six-month stock of raw materials through the mobilization of national strategic reserves etc (except for dairy and beverage products industry, whose stock was ranged for zero to one month at most). When there is a shortage of available labour facing heatwaves, up to 25% of excess productivity is provided based on the highest values from previous studies^17^. Products can be fully mobile within global trade networks and national products can be substituted for each other.

Supplementary Figure **7** e-h is the moderate adaptation scenario, and parameters such as inventory and excess capacity are used in line with the facts and are more common in previous studies^17^. The producer stocks one quarter's inventory of raw materials. When there is a shortage of available labour, a maximum of 10% excess productivity is provided. Products are partially mobile within global trade networks but need time to adjust. The allocation strategies of the countries in the model will be gradually shifted to unaffected countries (rather than in one step). Products can be substituted for each country except for construction.

Supplementary Figure **7** i-l is the low adaptation scenario, with highest economic losses. The parameters of the low adaptation scenario are set as follows. Producer stocks one month of raw material inventory. When the available labour force is insufficient, excess productivity cannot be provided through additional adaptation measures. The substitutability of products between countries is low due to information lags and trade barriers. When country A experiences a shortage in the supply of upstream intermediate products, it is unable to obtain substitute products from other countries. This scenario setting is close to a traditional static IO, which would overestimate economic losses and is the upper limit of the assessment. In the figure for the Monte Carlo analysis (Fig.4), we do not show the results under this one setting, as their curves are clearly biased towards the other type of clustering.

**As shown in** **Extended Data Fig. 5, Supplementary Figure 7 and Supplementary Figure 8, firstly, the core finding of this study, that the global supply chain amplification effect leads to a non-linear increase in economic losses from heat stress, is robust to a large number of rigorous tests.** The difference is only that the point at which the exponential growth occurs changes with the adaptive setting. Supplementary Figure **7** e-h is the baseline scenario similar to the result in the main text, where parameters such as inventories and excess production capacity are used in line with the facts and are more common in previous studies. In the high adaptation scenario (Supplementary Figure **7** a-d), economic losses increase at a high rate from 2050 to 2060. This is because each country is assumed to have sufficient production stocks for vulnerable industries to maintain production for a short period of time even in the event of upstream supply disruptions. And when extreme heat is frequent, new adaptive technologies can be generated and widely used in time to offset the health and labour productivity losses of heat stress to some extent. In contrast, when the adaptation parameter is set at its lowest setting (Supplementary Figure **7** i-l), economic losses will rapidly increase in very near future. As for the signs and the signals (e.g. which countries and regions most affected, which sectors are most affected), country-scale vulnerability is very robust to changes in parameters other than trade structure. Changes in the structure of trade, such as the year of the MRIO table for the base period, can have a significant impact on specific regions whose position in GVC has change significantly between reference years (as shown Extended Data Fig. 4). Under the 2011 trade structure, for example, economic losses in Africa could hardly be transmitted to Asia. However, in 2014, with East Asian Southeast Asian countries such as China and India trading closely with Africa, the assessment of indirect losses in Southeast Asia improved significantly. This also illustrates how well our model captures the transmission of economic risks between trading partners.

We also employed a dynamic Computable General Equilibrium (CGE) model for a parallel assessment, as part of the robustness check of the ARIO results. Specifically, the CGE model we utilized is a G-RDEM with 10 regions and 10 sectors. G-RDEM is a well-designed CGE tool for long-term counterfactual analysis and economic baseline generation based on provided gross domestic product (GDP) and population projections. It has undergone various enhancements tailored for generating long-term scenarios and simulations^18^. It includes an implicitly directly additive demand system with non-linear Engel curves, incorporates debt accumulation from foreign savings, introduces sector-specific productivity changes, endogenously determines aggregate saving rates, and incorporates time-varying cost shares for value added and individual intermediates. The parameters for these relationships are estimated through econometric methods using the most recent available data or derived from published research.

We employed this model to assess the impacts of future heatwaves in a manner akin to the assessment performed by the ARIO model in this study. The evaluation outcomes from the CGE model are depicted in Supplementary Figure 8. As evident from the figure, the two models' evaluation results exhibit consistent trends and, to a certain extent, align in magnitude. While the ARIO model does not account for changes in future economic structure, the dynamic CGE model considers such variations. The comparison of these outcomes demonstrates, from one perspective, that "although neglecting changes in future economic structure could lead to some distortion in ARIO model assessment outcomes, this weakness in ARIO does not significantly impact the evaluation results." Two reasonable explanations are as follows: (1) Although the economic structure may evolve in the future, it does so based on the existing economic framework. While disregarding this change could introduce bias in the results, this change is relatively minor compared to the current structure. Thus, capturing the current structure is more crucial. (2) The most significant changes in the future occur in regions with relatively smaller current economic scales, such as Africa, which constitutes a limited portion of the global economic volume. These changes have a marginal effect on the overall global evaluation results. Both these aspects contribute to the rationale behind the alignment in magnitude between considering structural changes (dynamic CGE) and disregarding structural changes (ARIO). But the core conclusion of this study, that the global supply chain amplification effect leads to a non-linear increase in economic losses from heat stress, is robust to a large number of rigorous tests. As shown in Supplementary Figure 8, the indirect losses of the 10-region and 10-sector CGE model show a non-linear upward trend.

**Secondly, the results of our assessment are slightly higher than CGE modelling results of previous studies and within the confidence interval.** The simulations we ran for dynamic CGE (2.1%±0.4% in 2060) result in about 10% to 20% lower global GDP losses than ARIO (2.5%±0.7% in 2060). We are more confident about the results of this research in two respects. On the one hand, we are using the latest, multi-model CMIP6 meteorological data and SSP socio-economic dynamics data. This is a significant improvement over the results of most previous assessments based on CMIP5 and RCP single-line scenario settings. On the other hand, Koks et al.^19^ found that CGE-based models tend to underestimate economic losses, while static IO-based models tend to overestimate economic losses. The simulations we ran under dynamic CGE model did result in about 10% to 20% lower global GDP losses than ARIO model. The ARIO model we use improves the IO model by introducing adaptations that are closer to the actual results. More importantly, a growing number of studies have found that sector aggregating in the CGE and IO models can introduce significant errors (often underestimations) into the indirect loss assessment. Even when based on the same data, the results of sector/area summation can vary several times from those calculated before the summation. For the first time, our model assesses global economic losses at a scale of 141 regions, 65 sectors, which fixes the bias introduced by the aggregation of the world into 10 or so regions and sectors.

In summary, we find that the strong adaptation hypothesis (quantified in the model in terms of the parameters associated with adaptation or substitution) can significantly reduce economic losses in the short term, under low emission scenarios. However, adaptation strategies for long-term climate change stress, especially under high emission scenarios, are not effective in reducing economic losses on long time scales (beyond 2060).


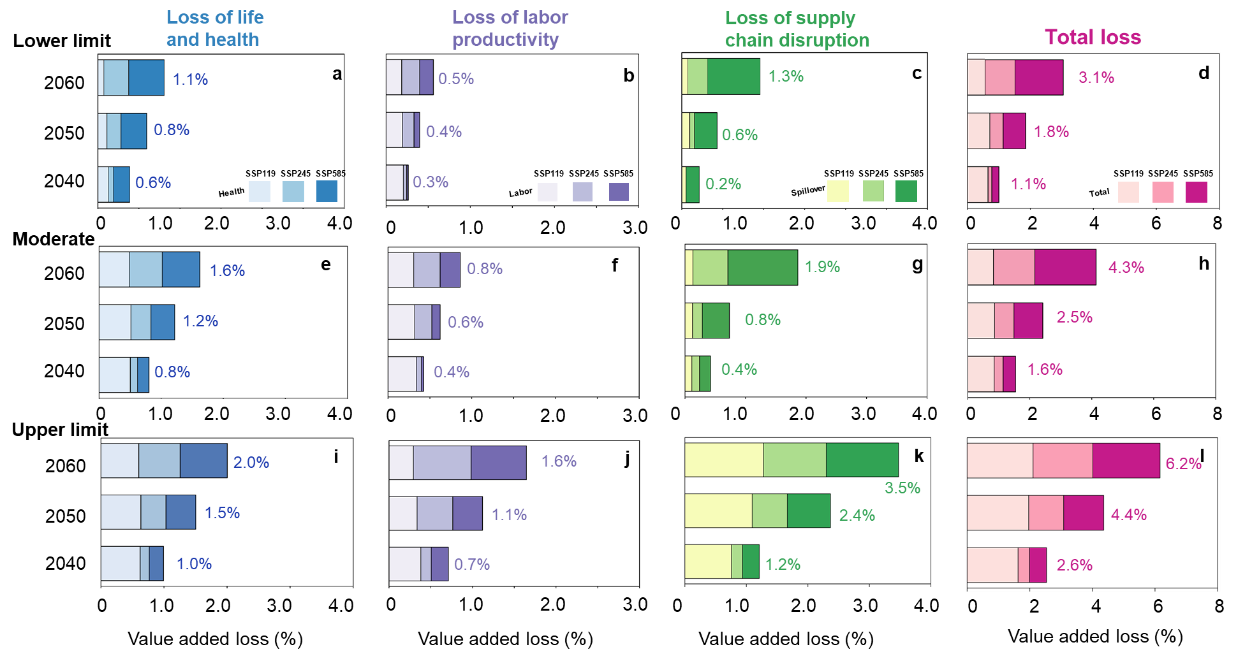


**Supplementary Figure 7 Evolutionary trends in global economic losses under combined parameter settings.** a-d shows the minimum value of the loss assessment for our range of uncertainty parameter settings under highest adaptation parameters. e-h shows the median value of the loss assessment for our range of uncertainty parameter settings. i-l shows the maximum value of the loss assessment for our range of uncertainty parameter settings under lowest adaptation parameters.


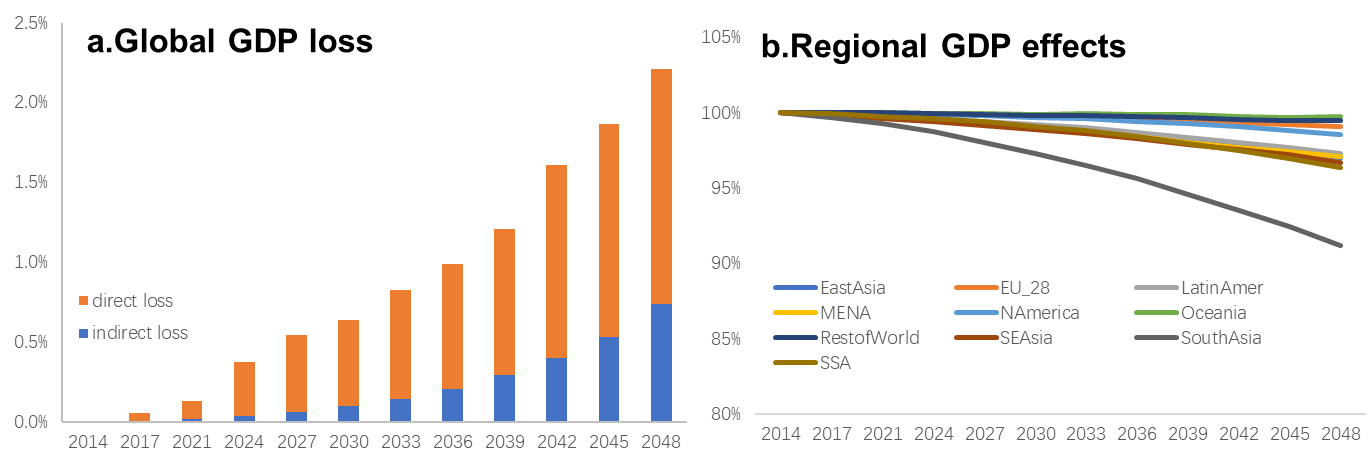


**Supplementary Figure 8 Global and regional economic losses for the SSP585 scenario under dynamic CGE simulation (10 region and 10 sector)**

In numerous experiments we have also found that indirect effects can not only work as risk amplifier but also as a risk reducer by means of trade rerouting or diversification as well as building up stocks. This reducer effect is particularly pronounced for countries with high risk in particular supply chains. For example, the indirect loss to Kyrgyzstan when trade substitution is not allowed is as high as 4.26% of GDP in 2060 under SSP85 scenario. When trade rerouting is allowed, the loss is reduced to 1.24%. At the global scale, trade rerouting and diversification in supply chains can reduce GDP losses by 10% to 15%. Building up reasonable and adequate stocks can delay and slightly reduce the impact of disaster shocks, reducing indirect losses by about 5% to 10% on a global scale.

In terms of validation, indirect losses are very difficult to investigate in statistical data. Our study does not aim predicting the true dynamics of GDP at regional or national levels because our economic impact model is focused on estimating the supply chain losses related to the heat stress assuming that other factors do not change, but the reality is that other factors will have changed, and the observed rates growth of GDP will be the net of all these changes. However, we can make some general comparisons of economic production with some anecdotal evidence, as well as empirical studies based on counterfactual assumptions.

We verify our results against realistic data by researching national statistical offices, reports, research articles, etc. as far as possible. Based on historical gridded meteorological observation data from the ERA5 reanalysis, we calculated the number of deaths and economic losses due to extreme heat for the years 1990-2022. Although economic losses cannot be directly compared, counterfactuals can be constructed by statistical and econometric means to capture the impact of heat stress on the GDP of the year. Drawing on the empirical statistical study published by Callahan & Mankin (2022) in *Science Advance*, we compared the actual economic losses measured by the ARIO (mechanistic model) and the econometric model. For example, based on subnational economic data and empirical regression methods, Callahan & Mankin found that Cumulative 1992–2013 losses from anthropogenic extreme heat fall between $5 trillion and $29.3 trillion (2010$) globally (average more than $16 trillion). In comparison, the evaluation results of our model are ranged from $15.0 trillion to $28.4 trillion (2010$) globally. Solomon (2010)^21^ used distributed-lag, autoregressive regression and found that in the Caribbean and Central America, output losses occurring in nonagricultural production (–2.4%/+1 °C) substantially exceed losses occurring in agricultural production (–0.1%/+1 °C), with 2.25% as indirect loss in the next year. In comparison, our results found that economic losses in the Caribbean and Central America from 1990-2010 were 1.97% (0.91%~2.39%) per degree. Interestingly, Solomon's research demonstrates that economic losses cannot be driven by direct losses in agriculture alone. It put forwards the significant impact of labour productivity, but fails to dissect the impact of indirect losses. In his research, wholesale, retail, restaurants and hotels show statistically significant responses to heat stress (−6.1%/+1 °C), but the reason was not clear as labour productivity was unlikely to be main driver of restaurant and hotel sectors (with low to medium intensity workload indoors and partially coverage with air-conditioning). Our assessment for accommodation, food and service activities sector in the Caribbean and Central America shows similar vulnerability (-3.5% to -5.7%/+1 °C), with backward and forward transmitted indirect losses (mainly due to decline of tourism demand, as well as supply shortage of food products) accounting for 60% of the total losses. The average annual direct loss from 1990 to 2010 in accommodation, food and service sector is 0.46% of sectoral VA while the indirect loss is 0.79%. An empirical study based on US state panel data from 1997 to 2011 similarly found a cascading effect due to extreme heat^22^. The article points out that the rise in average summer temperatures has a significant direct impact on labour productivity in the agricultural sector and, indirectly, in the food services and drinking sectors (-0.39% per °F) due to fluctuations in supply and demand. In comparison, the evaluation results of our model are (-0.28% per °F). This demonstrates that mechanism-based models and empirical models can complement each other.

In terms of news, anecdotes, or other study, our findings are not at all unprecedented in that studies on other past disasters have also shown that indirect effects can represent a significant share of total losses, or even dominate. For example, Sieg et al (2019) find that “The direct economic impacts of the flood event 2013 in Germany lie with a probability of 90% between 1.5 and 2.1 billion Euro, while the indirect economic impacts lie between 1.1 and 1.6 billion Euro. The ratios for Germany range between 0.7 and 0.9 indicating that indirect impacts can almost be as high as direct impacts”. In another study on the impacts of the 2011 earthquake in Japan, Inoue & Todo (2019), find that “Owing to the propagation of the shock, the total indirect effect is more than 100 times the total direct effect, or the total loss in the value added, of firms directly damaged by the earthquake.” In 2021, a large Bordeaux order was postponed at the last minute because the producer had no boxes in which to pack the wine (https://www.winemag.com/2021/10/25/wine-shipping-delays/). “There’s now a growing lack of materials for everything necessary for wine production, including glass, label inks, paper and cardboard”, said the export director for Bacalhôa Vinhos de Portugal.

Note that these comparisons are not rigorous because the distribution of direct losses caused by different disasters is different. For heat stress, anecdotes and news reveal the complex supply chain spillover effects. For example, the 2022 heatwave in Sichuan, China, has caused Toyota and Contemporary Amperex Technology, the world's largest battery maker, to suspend operations for one month. Numerous other manufacturers have also shut down shop during the blistering heat. SAIC Motor (China's largest automaker) and Tesla have also impacted operations in Shanghai, which is located far from Sichuan, because suppliers in the province have been unable to ship needed parts. At the macroeconomic level, uninsured losses from physical risks may affect resource availability and economic productivity across sectors with cascading impacts on the financial system. In report of Swiss Re Institute, they say “at the macroeconomic level, uninsured losses from physical risks (from climate change) may affect resource availability and economic productivity across sectors with cascading impacts on the financial system. Re/insurers should use their understanding of risk to help households, private companies and societies mitigate and adapt”. These cases show the cascading effect of heat stress through the supply chain.

In summary, our model has also proved to be a reasonable simulation for realistic losses for two reasons: (1) In terms of magnitude, our calculations capture the historical economic impact of severe heat stress in various countries (e.g. European heat waves of 2003 and 2015). The loss trends and regional-sectoral vulnerabilities are consistent with historical true losses (derived from the econometric model). The results are not strictly equivalent, but in the same order of magnitude. (2) In terms of mechanism, for the first time, our model reveals the mechanism by which economic losses are propagated, with indirect losses at each country-sector node (141 countries * 65 sectors = 9165 nodes) being traceable. Indirect losses for each node are traced backward or forward until the iteration reaches a node where 90% or more of the losses are direct losses. The traceable nature of our model makes it more reliable than “black-box” like models that cannot be traced, where only the total losses of the aggregated regions and sectors are reported and the impact often loses its practical significance for adaptation policy making. The model can therefore be optimised to a large extent close to reality when more real trade data for each node after shock is available in future works.

Considering the challenges of predicting changes to socioeconomic systems globally, we have followed the approach from the literature ^13,24–26^ to simulate supply chain indirect losses by considering the impact of future climate risks on current socioeconomic settings. We have not considered the potential substitution of labour with capital resulting from technological advances, such as mechanization. The results should therefore be interpreted with caution as indicating potential future climate change risks to the existing economy rather than as quantitative predictions, given that the static representation of the economic structure in our model inevitably skews the assessment in the long run. Our analysis ignores the different levels of trade openness and globalization among SSP narratives, as well as the role of dynamic factors such as technology and price. Again, although we have conducted robustness tests for different degrees of trade substitutability, the relevant parameter is set randomly in the Monte Carlo simulation rather than derived through a general equilibrium model. Moreover, we only include economic losses caused by heat stress on human activities without considering the impacts on infrastructure, crop growth, and other factors.

# Climate conditions in different scenarios


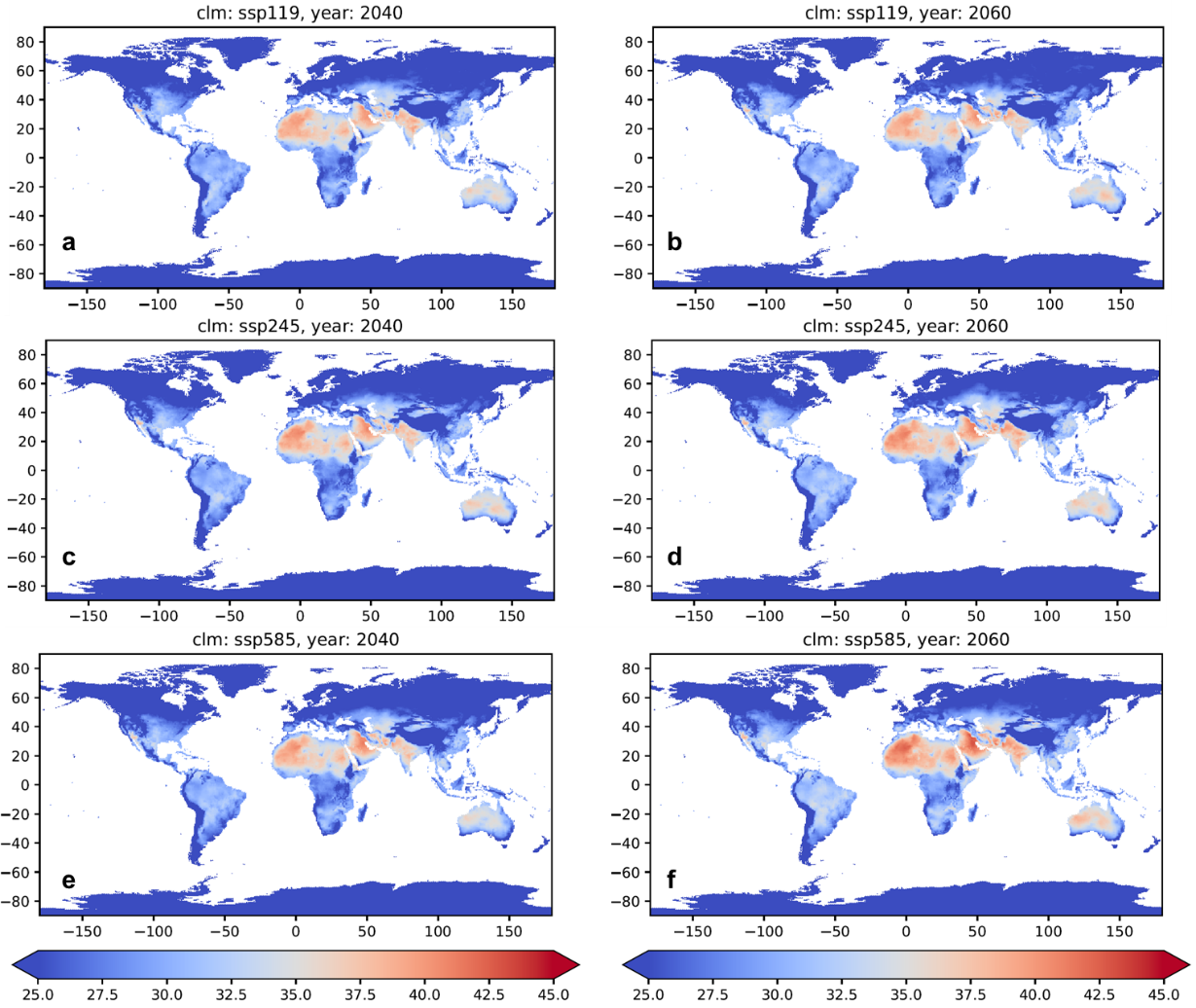


**Supplementary Figure 9 Maximum Daily Mean Near-Surface Air Temperature (K) under the SSP119 (a,b) , SSP245 (c, d) and SSP585 (e, f) scenarios in year 2040 (a,c,e) and 2060 (b,d,f).**

# Climate change vulnerability of economic losses by region


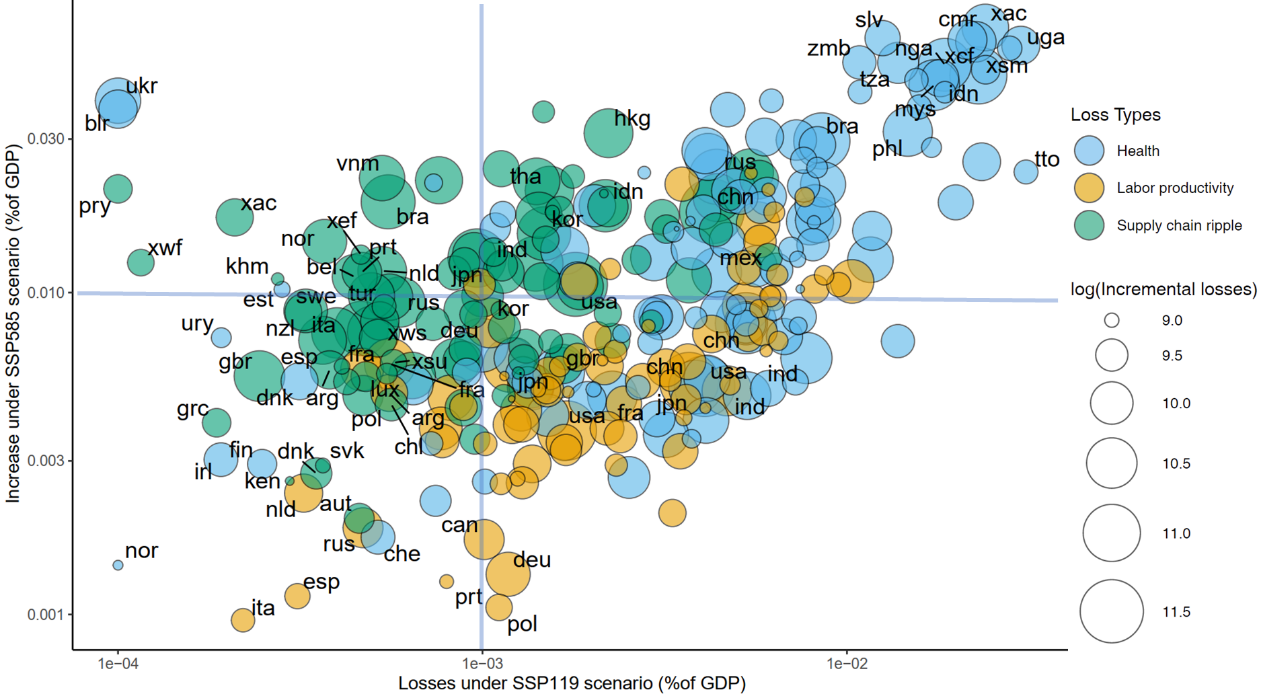


**Supplementary Figure 10 Loss patterns across regions in 2060 under SSP119 and SSP585 scenarios**.

The x-axis represents the percentage of economic loss in 2060 under the baseline SSP119 scenario and the y-axis represents the additional economic loss in the fastest warming SSP585 scenario, both using logarithmic scale. Losses of each country in are measured as a share of national GDP. Orange, blue and green represent labour productivity losses, health losses and indirect losses respectively. The size of the bubbles represents the size of the increase in loss from the SSP119 to the SSP585 scenario.

In terms of types of losses, direct labour productivity losses are relatively low as a proportion of GDP except for some least developed countries such as Malawi, Laos and Madagascar. Considerable labor losses are expected in the future for countries with persistent low incomes, as well as for outdoor labor. Because with economic development and higher GDP per capita in each country, more universal access to air conditioning has also significantly reduced potential future labour productivity losses. Health losses are relatively large and concentrated in south-central Africa, the Caribbean, and Eastern Europe. The distribution of supply chain cascading losses varies in the SSP119 scenario, but there is an overall rapid upward trend in the SSP585 scenario, with the incremental losses concentrated in the 1%-3% range of GDP.

In terms of loss patterns across different regions, Central and Southern Africa are suffering huge health losses in both scenarios due to increasing frequency of extreme heat waves and high base mortality rate. Several semi-periphery countries such as Brazil and Malaysia, have low losses under a low-warming SSP119 scenario, but surged up to 3% to 5% of GDP once a high emission SSP585 development path is adopted. Even several EU countries such as Greece, Norway and the United Kingdom which are less sensitive to mild climate change, have economic losses increased by nearly 1%, of which indirect losses are predominant. These are the countries that most need to rally the world around the most aggressive strategy to reduce GHG emissions. Several Central American countries are also sensitive to both climate change and scenario changes. Last but not least, this figure only shows the relative severity of economic losses from climate change, measured as a share of national GDP, across countries. The absolute losses for large economies are often huge. For example although labour and health losses in the US are a low percentage of the country's GDP, the absolute incremental economic losses ($) are equally large (over $100 billion) due to the size of the economy.

# Loss patterns in representative countries

Individual countries have different patterns of loss and vulnerability in the context of climate change. Based on the losses under the SSP119 scenario, the growth rate of losses over time and scenarios, and the sectors with the highest losses, we have conducted k-means (for continuous variables, e.g. percentage loss), EM algorithm (for ranking data, e.g. ranking of sectors with the highest percentage of losses) and k-Prototypes (for mixed variables, e.g. percentage loss, vulnerable sector category) clustering analysis of 141 regions. The k-means algorithm after the sector loss pattern quantification process performs best with minimized total within-cluster variation. GTAP 141 sectors were defined as 11 categories (1. GrainsCrops, 2. MeatLstk, 3. Extraction etc., based on official GTAP aggregation). National sectoral loss patterns are quantified as the number of occurrences in the top ten sectors of losses (Supplementary Table3). We use Elbow method to calculate the total within-cluster sum of square, and the appropriate number of clustering categories was determined to be 4. The clustering results are shown in Supplementary Figure **11**. According to the cluster centres, Cluster 1 is highly vulnerable to extreme heatwaves and health and labor productivity loss, with significant losses in sectors such as construction and agriculture under all the SSP119, SSP245 and SSP585 scenarios. Cluster 2 and suffers considerable both direct and indirect economic losses in SSP245 and SSP585 scenarios, with construction, agriculture, food processing and non-metallic manufacturing as the main sectors of loss. Cluster 3 is scenario change sensitive and these countries suffer almost no losses under the SSP119 scenario, but losses rise at a high rate under the SSP585 scenario. The main loss sector is metals and manufacturing. Cluster 4 is relatively resilient, with moderate levels of losses in all scenarios.

Supplementary Table3 The ten sectors with the highest losses per region (partial)

| Code | Reg | Sec1 | Sec2 | Sec3 | Sec4 | Sec5 | Sec6 | Sec7 | Sec8 | Sec9 | Sec10 |
| --- | --- | --- | --- | --- | --- | --- | --- | --- | --- | --- | --- |
| 1 | aus | 8 | 7 | 7 | 7 | 9 | 6 | 7 | 9 | 10 | 3 |
| 2 | nzl | 2 | 2 | 2 | 4 | 9 | 11 | 9 | 4 | 9 | 10 |
| 3 | xoc | 8 | 3 | 3 | 4 | 10 | 10 | 4 | 9 | 9 | 7 |
| 4 | chn | 1 | 3 | 8 | 10 | 6 | 9 | 7 | 4 | 7 | 6 |
| 5 | hkg | 3 | 5 | 4 | 8 | 4 | 7 | 6 | 6 | 6 | 7 |
| 6 | jpn | 8 | 10 | 7 | 6 | 4 | 10 | 7 | 6 | 10 | 10 |
| 7 | kor | 8 | 10 | 9 | 9 | 7 | 6 | 4 | 5 | 4 | 5 |
| 8 | mng | 4 | 3 | 2 | 9 | 2 | 2 | 7 | 2 | 5 | 8 |
| 9 | twn | 3 | 6 | 8 | 10 | 8 | 4 | 5 | 7 | 7 | 6 |
| 10 | xea | 8 | 2 | 7 | 3 | 3 | 4 | 9 | 9 | 9 | 9 |
| 11 | brn | 3 | 9 | 3 | 9 | 9 | 6 | 10 | 9 | 10 | 7 |
| 12 | khm | 10 | 8 | 1 | 3 | 3 | 1 | 2 | 1 | 1 | 4 |
| 13 | idn | 3 | 3 | 8 | 1 | 6 | 9 | 9 | 4 | 1 | 5 |
| 14 | lao | 1 | 3 | 5 | 4 | 4 | 8 | 5 | 2 | 11 | 1 |
| 15 | mys | 1 | 8 | 3 | 3 | 9 | 7 | 4 | 6 | 7 | 9 |
| 16 | phl | 3 | 7 | 8 | 4 | 7 | 5 | 4 | 2 | 5 | 10 |
| 17 | sgp | 7 | 6 | 4 | 6 | 8 | 7 | 10 | 8 | 7 | 9 |
| 18 | tha | 5 | 11 | 6 | 4 | 7 | 7 | 7 | 8 | 8 | 1 |
| 19 | vnm | 1 | 3 | 7 | 6 | 11 | 11 | 9 | 9 | 4 | 5 |
| 20 | xse | 3 | 10 | 3 | 1 | 8 | 2 | 3 | 1 | 1 | 8 |


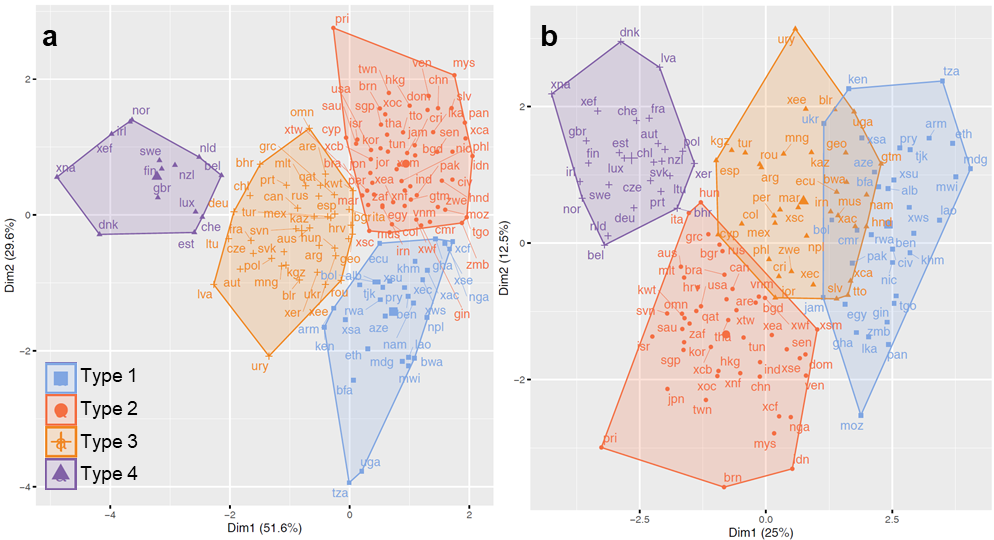


**Supplementary Figure 11 Cluster analysis of loss patterns in different regions.** Each point represents one of the 141 regions of GTAP. Principal component analysis (PCA) is performed and data points are plotted according to the first two principal components that explain the majority of the variance. **a**, the 3 highest loss sectors are included in the clusters. **b**, the 10 highest loss sectors are included in the clusters


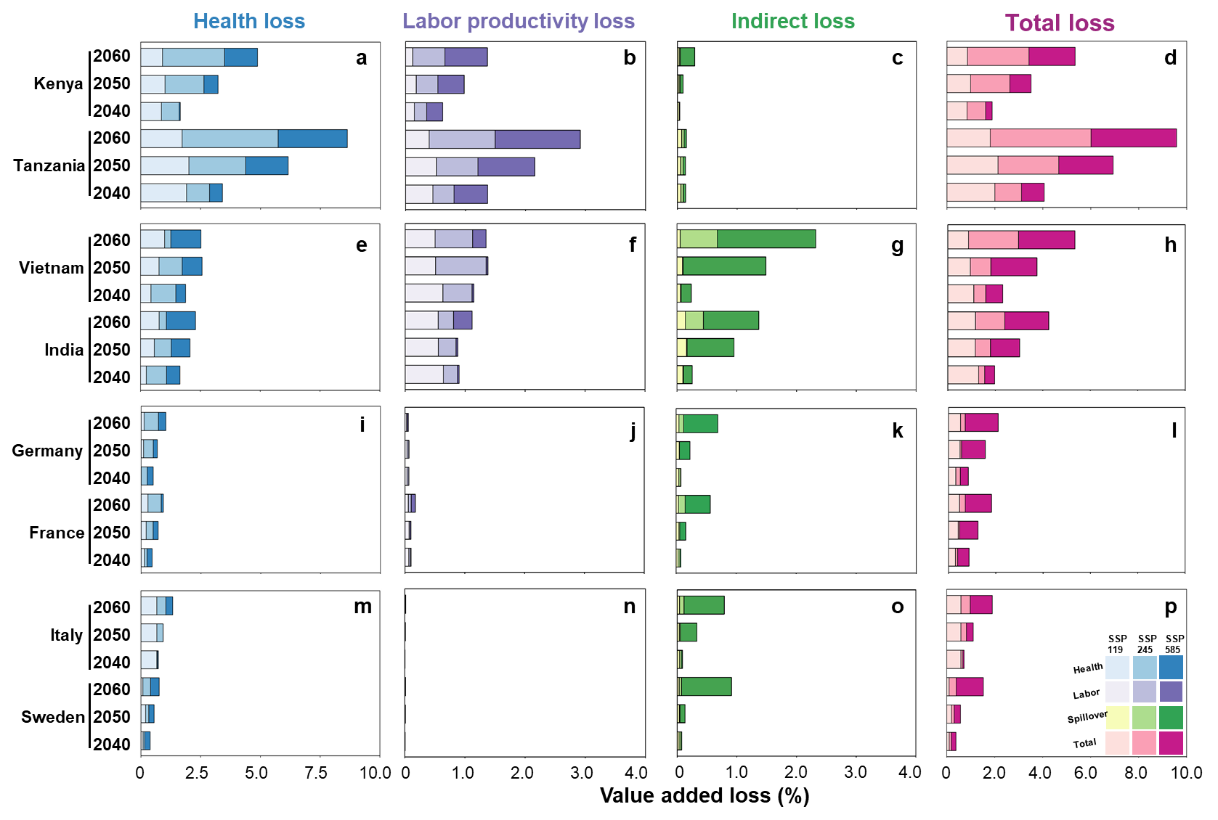


**Supplementary Figure 12 Evolution patterns of economic losses for representative countries. a-d**, loss patterns in type 1 countries, exemplified by **a-d** Kenya and Tanzania; **e-h**, type 2 countries, exemplified by Vietnam and India; **i-l**, type 3 countries, exemplified by Germany and France. **m-p**, type 4 countries, exemplified by Italy and Sweden.

Supplementary Figure 12 illustrates the evolution of the three loss types for representative countries in each region. Economic losses in most of African, South and South East Asian countries are dominated by direct losses of health and labour productivity. West Africa is already hot and climate change can lead to a much higher risk of average summer temperatures exceeding the human body's tolerance range. However, sudden temperature change is less frequent in the region and the frequency of extreme heat waves and their mortality is not high. South and Southeast Asian, and some of Latin American countries suffered considerable economic losses in all three categories, resulting in their total economic losses being among the highest in the world. Climate change has led to both a rapid increase in average daily summer temperatures and a significant increase in the probability of extreme heat waves. Further, South Asian countries are at a stage of development where they are integrating into global value chains. The cascading effect of disruptions in the chains is also causing economic losses of 1.5% to 3.5% for Vietnam and India. Economic losses in Western European countries are dominated by health and cascade effects and increase exponentially in high warming scenarios. The relatively mild summer temperatures in Western Europe, combined with the more than adequate penetration of indoor air conditioning, have resulted in lower losses of labour productivity. But sudden and extreme heat waves are already not uncommon in Europe, and climate change will significantly increase health losses (1%~2% of GDP). In addition, Western European countries import raw materials from South East Asia, the Middle East and other regions, and cascading effects are the dominant losses in the high warming scenario. Under SSP119 scenario, Western European countries are well protected from cascading effects in GSCs through intra-regional trade complementarities. However, the supply chain imbalances in the SSP585 scenario exceed the adaptation threshold and losses rise sharply to 3-3.5 per cent.

# Sectoral loss patterns in different types of countries

As shown in Fig.3 and Extended Data Fig. 9, the crop farming, construction and mining sectors suffered the most in most countries, especially in several African and Asian countries whose economy depends on primary industries. Zimbabwe and Malawi also fall into this category. These countries are characterised by a very hot climate and a national economy that is more dependent on the primary sector. In addition, the low level of air conditioning makes some indoor work and even service industries suffer from heat waves. Malawi's tourism, chemical manufacturing and trade sectors, for example, have experienced significant declines in labour productivity. While labour losses are the core economic loss in such countries, cascading losses over time, especially under the high warming SSP585 scenario, are not insignificant in less developed countries. Cascading losses in sectors such as electronics manufacturing and restaurants may even surpass labour losses as the largest source of losses.

Countries in type 2 tend to include emerging economies at low and medium latitudes. Heat-induced economic losses in these economies, represented by India, are concentrated in the primary sector, construction, non-metallic and metal manufacturing, chemicals and other manufacturing industries. On the one hand, these countries are located at mid-latitudes and some industries are severely affected by extreme heat. On the other hand, these countries are closely involved in international trade and the cascade effect is significant in industries such as non-metallic products, ferrous metals and some plastic products.

Countries in type 3 include Western European countries such as Germany and France, as well as mid- to high-latitude countries in the Southern Hemisphere such as Australia. Labor productivity losses in these countries are lower than in the low and middle latitude countries and are usually limited to the construction industry (and mining in Australia). The economic loss for each sector is less than 1% in the SSP119 scenario. However, indirect losses are significant in the metal smelting, metal products, and beverage and tobacco industries.

The countries in type 4 tend to be developed countries in the highest latitudes of northern Europe, represented by Sweden. The sectors in this group of countries suffer virtually negligible labour productivity or health losses under both SSP119 and SSP245 scenarios but there is a significant increase in 2060 under the SSP585 scenario. Some medium- and high-end equipment manufacturing industries were significantly affected by indirect losses. For example, electrical equipment in the United Kingdom suffered a 2.2 per cent loss in value added. In particular, Norwegian energy products such as Petroleum and coal products show an increase in the loss index to 2.3% in the high warming scenario. Similarly, chemical products in the United Kingdom have a loss of 2.5 per cent.

Overall, on a global scale, economic losses are characterized by a “ladder” shape from low to high latitudes and from upstream to downstream in the global value chain. There seems to be a link between the gradient in latitude of individual countries, the gradient in sectoral economic losses and the position of the value chain of each country and sector. The clustering of countries shows how economic losses propagate along the global value chain from the low latitudes, which are hardest hit by warming, to the high latitudes, which have cooler climates.

# Disaggregated impacts of climate change and population dynamics

By fixing the population grid (number and distribution) in current situation in year 2020, we decompose the respective contributions of climate change and population dynamics to global economic losses. As population size is only used as a weighted indicator in our model, only the spatial distribution of the population affects the assessment results, not the population size. We found that about 97% of global economic losses in this study are explained by climate change. Dynamic population scenario shows a slight increase of 0.11% in global economic losses compared to the fixed population scenario. The effects of spatial demographic change make a relatively weak contribution in individual countries. In Nepal, Pakistan and Niger, for example, population dynamics increase direct losses by an additional 0.7%, 0.4% and 0.4% of country’s GDP respectively. This suggests that the spatial expansion of future populations in Nepal and Pakistan (i.e. the process of urbanisation) is sensitive to climate change risk and exposes greater proportion of the population to heat stress. Population dynamics in Qatar and UAE have reduced national GDP losses by 0.3% and 0.2%. This suggests that Qatar and the UAE are at low climate risk for future urbanisation or spatial distribution of population.

The population distribution in the existing SSP scenario does not consider preferences for future climate livability under the effects of climate change and climate-induced migration. Therefore future studies of urban expansion or population growth should pay more attention to climatic factors. Spatial population dynamics have the potential to reduce global climate risks if climate migration, risk aversion and other factors are fully considered.


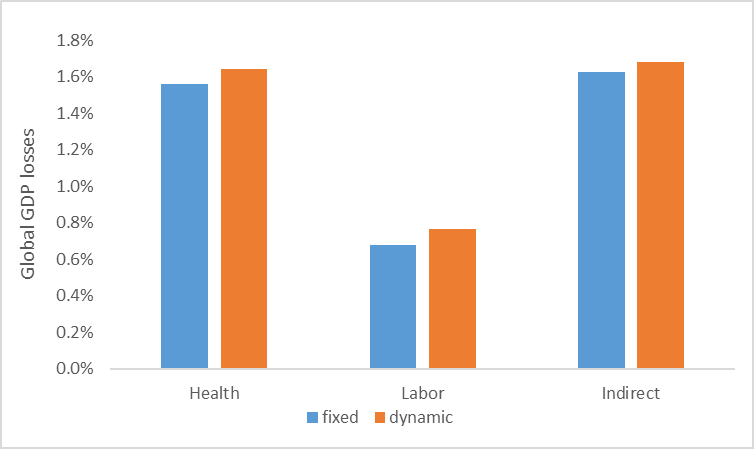


**Supplementary Figure 13 Economic losses from global heat stress under fixed and dynamic populations, under SSP585 scenario 2060.**

# Comparison of IO based and CGE based modellings

Many methods have been proposed to analyze the propagation of negative shocks through economic networks in the existing literature, which can be mainly categorized into two strands. The first category comprises the models based on Input-Output (IO) analysis. A standard IO model can be described as a static linear model that presents the economy through sets of fixed relationships between sectors themselves (the producers) and others (the consumers). Therefore, IO models can capture the ripple effect triggered by supply constraints in the economic networks in a very straightforward way, which makes it one of the most applied models to assess the indirect economic impacts of disasters.

The second type of model widely used in disaster impact assessment is Computable General Equilibrium (CGE) based models. A neoclassical CGE model is a system of equations that describes the behavior of firms and households and their interactions by functional relationships subject to prices and market clearing conditions. Compared with IO models, CGE models consider the strategic behaviors of economic agents under the profit and utility maximization assumptions. CGE models, therefore, can better capture the adaptive response of economic agents to shocks. However, due to their relative-prices adjustment mechanism, CGE models tend to be overly optimistic about market flexibility in disaster aftermath and underestimate the losses as it allows extreme flexibility for no-cost substitution ^19^.

In this research, we employed both ARIO and dynamic CGE model to assess the impacts of future heatwaves. As evident from the results, the two models' evaluation results exhibit consistent trends and, to a certain extent, align in magnitude. While the ARIO model does not account for changes in future economic structure, the dynamic CGE model considers such variations. The comparison of these outcomes demonstrates, from one perspective, that "although neglecting changes in future economic structure could lead to some distortion in ARIO model assessment outcomes, this weakness in ARIO does not significantly impact the evaluation results." Two reasonable explanations are as follows: (1) Although the economic structure may evolve in the future, it does so based on the existing economic framework. While disregarding this change could introduce bias in the results, this change is relatively minor compared to the current structure. Thus, capturing the current structure is more crucial. (2) The most significant changes in the future occur in regions with relatively smaller current economic scales, such as Africa, which constitutes a limited portion of the global economic volume. These changes have a marginal effect on the overall global evaluation results. Both these aspects contribute to the rationale behind the alignment in magnitude between considering structural changes (dynamic CGE) and disregarding structural changes (ARIO).

Hence, IO models offer a more flexible framework to simulate such out-of-equilibrium dynamics. With their more rigid structure, IO models are often considered more suitable for post-disaster supply-chain disaster risk assessments. We choose ARIO model in this research mainly for three reasons. Firstly, ARIO increases adaptability and avoids loss overestimation compared to traditional static IO models. And with much higher sectoral and regional resolution than the CGE model, it avoids considerable errors due to aggregation^27–29^. Secondly, CGE models are often considered less suitable for disaster assessments compared to Input-Output (IO) models due to their potentially overly optimistic flexibility in post-disaster markets. Thirdly, although dynamic CGE models can consider structural changes, they involve numerous parameters, potentially introducing additional uncertainties.

Supplementary Table 4 Descriptions of the 65 Sectors of GTAP 10

| SID | Code | Name | Characteristics |
| --- | --- | --- | --- |
| 1 | pdr | Paddy rice | GrainsCrops |
| 2 | wht | Wheat | GrainsCrops |
| 3 | gro | Cereal grains nec | GrainsCrops |
| 4 | v_f | Vegetables, fruit, nuts | GrainsCrops |
| 5 | osd | Oil seeds | GrainsCrops |
| 6 | c_b | Sugar cane, sugar beet | GrainsCrops |
| 7 | pfb | Plant-based fibers | GrainsCrops |
| 8 | ocr | Crops nec | GrainsCrops |
| 9 | ctl | Cattle, sheep and goats, horses | MeatLstk |
| 10 | oap | Animal products nec | MeatLstk |
| 11 | rmk | Raw milk | MeatLstk |
| 12 | wol | Wool, silk-worm cocoons | MeatLstk |
| 13 | frs | Forestry | Extraction |
| 14 | fsh | Fishing | Extraction |
| 15 | coa | Coal | Extraction |
| 16 | oil | Oil | Extraction |
| 17 | gas | Gas | Extraction |
| 18 | oxt | Other Extraction | Extraction |
| 19 | cmt | Meat: cattle, sheep, goats, horse | MeatLstk |
| 20 | omt | Meat products nec | MeatLstk |
| 21 | vol | Vegetable oils and fats | ProcFood |
| 22 | mil | Dairy products | ProcFood |
| 23 | pcr | Processed rice | GrainsCrops |
| 24 | sgr | Sugar | ProcFood |
| 25 | ofd | Food products nec | ProcFood |
| 26 | b_t | Beverages and tobacco products | ProcFood |
| 27 | tex | Textiles | TextWapp |
| 28 | wap | Wearing apparel | TextWapp |
| 29 | lea | Leather products | LightMnfc |
| 30 | lum | Wood products | LightMnfc |
| 31 | ppp | Paper products, publishing | LightMnfc |
| 32 | p_c | Petroleum, coal products | HeavyMnfc |
| 33 | chm | Chemical products | HeavyMnfc |
| 34 | bph | Basic pharmaceutical products | HeavyMnfc |
| 35 | rpp | Rubber and plastic products | HeavyMnfc |
| 36 | nmm | Mineral products nec | HeavyMnfc |
| 37 | i_s | Ferrous metals | HeavyMnfc |
| 38 | nfm | Metals nec | HeavyMnfc |
| 39 | fmp | Metal products | LightMnfc |
| 40 | ele | Computer, electronic and optical products | HighTech |
| 41 | eeq | Electrical equipment | HighTech |
| 42 | ome | Machinery and equipment nec | HighTech |
| 43 | mvh | Motor vehicles and parts | HighTech |
| 44 | otn | Transport equipment nec | LightMnfc |
| 45 | omf | Manufactures nec | LightMnfc |
| 46 | ely | Electricity | Util_Cons |
| 47 | gdt | Gas manufacture, distribution | Util_Cons |
| 48 | wtr | Water | Util_Cons |
| 49 | cns | Construction | Util_Cons |
| 50 | trd | Trade | TransComm |
| 51 | afs | Accommodation, Food and service activities | TransComm |
| 52 | otp | Transport nec | TransComm |
| 53 | wtp | Water transport | TransComm |
| 54 | atp | Air transport | TransComm |
| 55 | whs | Warehousing and support activities | TransComm |
| 56 | cmn | Communication | TransComm |
| 57 | ofi | Financial services nec | OthServices |
| 58 | ins | Insurance | OthServices |
| 59 | rsa | Real estate activities | OthServices |
| 60 | obs | Business services nec | OthServices |
| 61 | ros | Recreational and other services | OthServices |
| 62 | osg | Public Administration and defense | OthServices |
| 63 | edu | Education | OthServices |
| 64 | hht | Human health and social work activities | OthServices |
| 65 | dwe | Dwellings | OthServices |

Supplementary Table 5 Description of the 141 regions of GTAP 10

| RID | Code | Name |
| --- | --- | --- |
| 1 | aus | Australia |
| 2 | nzl | New Zealand |
| 3 | xoc | Rest of Oceania |
| 4 | chn | China |
| 5 | hkg | Hong Kong |
| 6 | jpn | Japan |
| 7 | kor | Korea Republic of |
| 8 | mng | Mongolia |
| 9 | twn | Taiwan |
| 10 | xea | Rest of East Asia |
| 11 | brn | Brunei Darussalam |
| 12 | khm | Cambodia |
| 13 | idn | Indonesia |
| 14 | lao | Lao Peoples Democratic Republic |
| 15 | mys | Malaysia |
| 16 | phl | Philippines |
| 17 | sgp | Singapore |
| 18 | tha | Thailand |
| 19 | vnm | Viet Nam |
| 20 | xse | Rest of Southeast Asia |
| 21 | bgd | Bangladesh |
| 22 | ind | India |
| 23 | npl | Nepal |
| 24 | pak | Pakistan |
| 25 | lka | Sri Lanka |
| 26 | xsa | Rest of South Asia |
| 27 | can | Canada |
| 28 | usa | United States of America |
| 29 | mex | Mexico |
| 30 | xna | Rest of North America |
| 31 | arg | Argentina |
| 32 | bol | Bolivia |
| 33 | bra | Brazil |
| 34 | chl | Chile |
| 35 | col | Colombia |
| 36 | ecu | Ecuador |
| 37 | pry | Paraguay |
| 38 | per | Peru |
| 39 | ury | Uruguay |
| 40 | ven | Venezuela |
| 41 | xsm | Rest of South America |
| 42 | cri | Costa Rica |
| 43 | gtm | Guatemala |
| 44 | hnd | Honduras |
| 45 | nic | Nicaragua |
| 46 | pan | Panama |
| 47 | slv | El Salvador |
| 48 | xca | Rest of Central America |
| 49 | dom | Dominican Republic |
| 50 | jam | Jamaica |
| 51 | pri | Puerto Rico |
| 52 | tto | Trinidad and Tobago |
| 53 | xcb | Caribbean |
| 54 | aut | Austria |
| 55 | bel | Belgium |
| 56 | bgr | Bulgaria |
| 57 | hrv | Croatia |
| 58 | cyp | Cyprus |
| 59 | cze | Czech Republic |
| 60 | dnk | Denmark |
| 61 | est | Estonia |
| 62 | fin | Finland |
| 63 | fra | France |
| 64 | deu | Germany |
| 65 | grc | Greece |
| 66 | hun | Hungary |
| 67 | irl | Ireland |
| 68 | ita | Italy |
| 69 | lva | Latvia |
| 70 | ltu | Lithuania |
| 71 | lux | Luxembourg |
| 72 | mlt | Malta |
| 73 | nld | Netherlands |
| 74 | pol | Poland |
| 75 | prt | Portugal |
| 76 | rou | Romania |
| 77 | svk | Slovakia |
| 78 | svn | Slovenia |
| 79 | esp | Spain |
| 80 | swe | Sweden |
| 81 | gbr | United Kingdom |
| 82 | che | Switzerland |
| 83 | nor | Norway |
| 84 | xef | Rest of EFTA |
| 85 | alb | Albania |
| 86 | blr | Belarus |
| 87 | rus | Russian Federation |
| 88 | ukr | Ukraine |
| 89 | xee | Rest of Eastern Europe |
| 90 | xer | Rest of Europe |
| 91 | kaz | Kazakhstan |
| 92 | kgz | Kyrgyzstan |
| 93 | tjk | Tajikistan |
| 94 | xsu | Rest of Former Soviet Union |
| 95 | arm | Armenia |
| 96 | aze | Azerbaijan |
| 97 | geo | Georgia |
| 98 | bhr | Bahrain |
| 99 | irn | Iran Islamic Republic of |
| 100 | isr | Israel |
| 101 | jor | Jordan |
| 102 | kwt | Kuwait |
| 103 | omn | Oman |
| 104 | qat | Qatar |
| 105 | sau | Saudi Arabia |
| 106 | tur | Turkey |
| 107 | are | United Arab Emirates |
| 108 | xws | Rest of Western Asia |
| 109 | egy | Egypt |
| 110 | mar | Morocco |
| 111 | tun | Tunisia |
| 112 | xnf | Rest of North Africa |
| 113 | ben | Benin |
| 114 | bfa | Burkina Faso |
| 115 | cmr | Cameroon |
| 116 | civ | Cote dIvoire |
| 117 | gha | Ghana |
| 118 | gin | Guinea |
| 119 | nga | Nigeria |
| 120 | sen | Senegal |
| 121 | tgo | Togo |
| 122 | xwf | Rest of Western Africa |
| 123 | xcf | Central Africa |
| 124 | xac | South Central Africa |
| 125 | eth | Ethiopia |
| 126 | ken | Kenya |
| 127 | mdg | Madagascar |
| 128 | mwi | Malawi |
| 129 | mus | Mauritius |
| 130 | moz | Mozambique |
| 131 | rwa | Rwanda |
| 132 | tza | Tanzania United Republic of |
| 133 | uga | Uganda |
| 134 | zmb | Zambia |
| 135 | zwe | Zimbabwe |
| 136 | xec | Rest of Eastern Africa |
| 137 | bwa | Botswana |
| 138 | nam | Namibia |
| 139 | zaf | South Africa |
| 140 | xsc | Rest of South African Customs Union |
| 141 | xtw | Rest of the World |

Supplementary Table 6 compiled heat stress cost information for validation of health losses

| Country | Year | Total Deaths | 95 low | 95 mid | 95 top | 97.5 low | 97.5 mid | 97.5 top |
| --- | --- | --- | --- | --- | --- | --- | --- | --- |
| Australia | 2009 | 347 | 200.7131 | 258.5412 | 316.985 | 95.57662 | 121.9176 | 148.8487 |
| Australia | 2014 | 139 | 152.0082 | 196.5776 | 241.6421 | 69.70822 | 88.54469 | 107.7456 |
| Austria | 2003 | 345 | 336.9842 | 442.5227 | 549.3438 | 237.1911 | 288.7236 | 340.8239 |
| Belgium | 2003 | 1175 | 255.1225 | 335.0232 | 415.8949 | 248.2138 | 302.141 | 356.6624 |
| Belgium | 2006 | 940 | 371.9725 | 488.4689 | 606.3809 | 453.0887 | 551.5273 | 651.0506 |
| Belgium | 2015 | 410 | 131.0216 | 172.0556 | 213.5883 | 107.1257 | 130.3999 | 153.9306 |
| Belgium | 2019 | 400 | 198.4938 | 260.6591 | 323.5799 | 231.0133 | 281.2035 | 331.9468 |
| Belgium | 2020 | 1460 | 275.2344 | 361.4338 | 448.6807 | 240.1918 | 292.3761 | 345.1355 |
| Canada | 2021 | 815 | 708.0234 | 927.5004 | 1149.599 | 537.6459 | 655.2342 | 774.1683 |
| Croatia | 2003 | 788 | 311.0527 | 398.7893 | 487.3994 | 190.9114 | 243.1523 | 296.6623 |
| Czech Republic (the) | 2003 | 418 | 355.2726 | 466.5388 | 579.1571 | 164.3367 | 200.0407 | 236.1381 |
| France | 2003 | 19490 | 2251.613 | 2933.964 | 3624.152 | 2236.122 | 2758.189 | 3288.291 |
| France | 2006 | 1388 | 1814.071 | 2367.991 | 2928.358 | 1848.195 | 2271.901 | 2701.697 |
| France | 2015 | 3275 | 1423.583 | 1856.281 | 2293.975 | 1145.206 | 1415.341 | 1689.775 |
| France | 2019 | 868 | 1683.011 | 2192.616 | 2708.067 | 1690.492 | 2086.442 | 2488.434 |
| France | 2020 | 1924 | 1419.694 | 1851.799 | 2288.907 | 1101.285 | 1356.756 | 1616.012 |
| France | 2022 | 2816 | 2613.139 | 3402.799 | 4201.482 | 2270.287 | 2815.096 | 3369.205 |
| Germany | 2003 | 9355 | 2896.331 | 3803.42 | 4721.532 | 2575.142 | 3134.621 | 3700.264 |
| Germany | 2022 | 4500 | 2445.732 | 3211.7 | 3986.976 | 1351.963 | 1645.692 | 1942.658 |
| Hungary | 2007 | 500 | 440.3874 | 565.3037 | 691.4791 | 342.5269 | 435.9572 | 531.642 |
| India | 2002 | 1030 | 7922.652 | 10921.6 | 13996.47 | 3545.951 | 5443.798 | 7369.908 |
| India | 2015 | 2248 | 10542.95 | 14538.6 | 18635.63 | 6014.157 | 9297.777 | 12630.04 |
| Italy | 2003 | 20089 | 4419.347 | 5665.905 | 6924.889 | 3903.023 | 5018.122 | 6160.574 |
| Netherlands (the) | 2003 | 965 | 328.2475 | 431.0499 | 535.1015 | 189.7805 | 231.0125 | 272.6987 |
| Netherlands (the) | 2006 | 1000 | 477.0061 | 626.3975 | 777.6043 | 627.7613 | 764.1495 | 902.0405 |
| Netherlands (the) | 2019 | 400 | 257.997 | 338.7979 | 420.5807 | 309.6258 | 376.8955 | 444.9064 |
| Netherlands (the) | 2020 | 400 | 307.2215 | 403.4389 | 500.8254 | 354.6843 | 431.7434 | 509.6516 |
| Pakistan | 2015 | 1229 | 1162.88 | 1596.165 | 2040.076 | 310.2804 | 474.3978 | 640.945 |
| Pakistan | 2018 | 180 | 2190.328 | 2992.882 | 3814.459 | 818.5973 | 1224.624 | 1636.834 |
| Portugal | 2003 | 2696 | 287.3821 | 371.3314 | 456.1807 | 237.2281 | 299.1597 | 362.3749 |
| Portugal | 2005 | 462 | 223.9115 | 289.3728 | 355.537 | 121.2801 | 151.0054 | 181.2602 |
| Portugal | 2022 | 1063 | 201.349 | 260.6122 | 320.5204 | 101.4632 | 128.6051 | 156.3187 |
| Russian Federation (the) | 2001 | 276 | 4147.827 | 5410.227 | 6687.238 | 3272.803 | 4038.754 | 4816.571 |
| Russian Federation (the) | 2010 | 55736 | 9171.178 | 11973.77 | 14809.03 | 12196.91 | 14980.49 | 17802.88 |
| Slovenia | 2003 | 289 | 115.1388 | 150.4814 | 186.2392 | 93.97239 | 115.7808 | 137.9192 |
| Spain | 2003 | 15090 | 1359.359 | 1757.756 | 2160.466 | 1103.319 | 1424.306 | 1751.598 |
| Spain | 2022 | 4655 | 2031.409 | 2621.437 | 3217.738 | 2047.087 | 2648.332 | 3262.125 |
| Switzerland | 2003 | 1039 | 284.0649 | 373.03 | 463.0761 | 287.7469 | 350.2631 | 413.4683 |
| United Kingdom of Great Britain and Northern Ireland (the) | 2003 | 301 | 1218.082 | 1599.568 | 1985.689 | 1225.424 | 1491.661 | 1760.832 |
| United Kingdom of Great Britain and Northern Ireland (the) | 2013 | 760 | 1188.395 | 1560.583 | 1937.294 | 956.3729 | 1164.156 | 1374.228 |
| United Kingdom of Great Britain and Northern Ireland (the) | 2020 | 2556 | 955.6915 | 1255 | 1557.946 | 753.2991 | 916.9617 | 1082.428 |
| United Kingdom of Great Britain and Northern Ireland (the) | 2022 | 3271 | 1898.015 | 2492.445 | 3094.099 | 1720.069 | 2093.774 | 2471.596 |

Supplementary Table 7 Compiled heat stress cost information for validation of economic losses (partial)

| Time | Region | Economic loss/ reported death |
| --- | --- | --- |
| 1980-2000 | 32 European countries | $71 billion |
| 2020 | U.S. Worker Productivity Loss | $100 billion |
| 2022 | China | $400 million |
| 2003 | Europe | 15 billion euros |
| 1980-2020 | U.S. | - |
| 2018, 2019 | German Agriculture + Industry and Commerce | 25.6 billion euros + 9 billion euros |
| 2017 | Global | 670 billion$ |
| 2030 | Global Countries |  |
| 2001-2020 | Global | 2.1 trillion in 2017 PPP$ |
| 2030、2050、2100 | Global, China, EU, India, Russia, US | See Table6 in paper |
| 2020-2100 | high-income countries and low-income countries | See Figure1 in paper |
| 1961-2010；1991-2010 Cumulative | Global | See SI by each country |
| 2020-2100 | Global | See Figure4 in paper |
| 1992–2013 | Global | $16 to $50 trillion |
| 2010 | Russia | $15 billion |
| 1980-2000 | 32 European countries | $71 billion |
| 2030 | India | 4.5 % GDP $15 billion to $25 billion |
| 2015 | India | 2.4 % GDP |
| 2003 2010 | European | 0.3–0.5% of GDP |
| 2010 | USA Automobile industry | 1.5% of Sectoral production |
| 2070-2090 | USA | 0.2% to 0.4% in low emission scenario, 1.2% in high emission scenario |
| 1997-2011 samples | USA Food services and drinking places | 0.39% per °F due to propagation effect |
| 1997-2012 samples | USA retail and wholesale sectors | 0.24% to 0.28% in the output growth |

# Reference

1. Guo, Y. *et al.* Heat Wave and Mortality: A Multicountry, Multicommunity Study. *Environmental Health Perspectives* **125**, 087006 (2017).

2. CRED / UCLouvain. Database | EM-DAT.

3. Hersbach, H. *et al.* The ERA5 global reanalysis. *Quarterly Journal of the Royal Meteorological Society* **146**, 1999–2049 (2020).

4. Center For International Earth Science Information Network-CIESIN-Columbia University. Gridded Population of the World, Version 4 (GPWv4): Population Count, Revision 11. (2018) doi:10.7927/H4JW8BX5.

5. Sera, F. *et al.* Air Conditioning and Heat-related Mortality: A Multi-country Longitudinal Study. *Epidemiology* **31**, 779 (2020).

6. Benmarhnia, T. *et al.* A Difference-in-Differences Approach to Assess the Effect of a Heat Action Plan on Heat-Related Mortality, and Differences in Effectiveness According to Sex, Age, and Socioeconomic Status (Montreal, Quebec). *Environmental Health Perspectives* **124**, 1694–1699 (2016).

7. Benmarhnia, T., Deguen, S., Kaufman, J. S. & Smargiassi, A. Vulnerability to Heat-related Mortality: A Systematic Review, Meta-analysis, and Meta-regression Analysis. *Epidemiology* **26**, 781 (2015).

8. The human cost of disasters: an overview of the last 20 years (2000-2019) | UNDRR. http://www.undrr.org/publication/human-cost-disasters-overview-last-20-years-2000-2019 (2020).

9. Han, Q., Sun, S., Liu, Z., Xu, W. & Shi, P. Accelerated exacerbation of global extreme heatwaves under warming scenarios. *International Journal of Climatology* **42**, 5430–5441 (2022).

10. Cheng, J. *et al.* Heatwave and elderly mortality: An evaluation of death burden and health costs considering short-term mortality displacement. *Environment International* **115**, 334–342 (2018).

11. Wondmagegn, B. Y. *et al.* Impact of heatwave intensity using excess heat factor on emergency department presentations and related healthcare costs in Adelaide, South Australia. *Science of The Total Environment* **781**, 146815 (2021).

12. Zhang, L. *et al.* Mortality effects of heat waves vary by age and area: a multi-area study in China. *Environ Health* **17**, 54 (2018).

13. García-León, D. *et al.* Current and projected regional economic impacts of heatwaves in Europe. *Nat Commun* **12**, 5807 (2021).

14. Orlov, A., Sillmann, J., Aunan, K., Kjellstrom, T. & Aaheim, A. Economic costs of heat-induced reductions in worker productivity due to global warming. *Global Environmental Change* **63**, 102087 (2020).

15. Casanueva, A. *et al.* Climate projections of a multivariate heat stress index: the role of downscaling and bias correction. *Geoscientific Model Development* **12**, 3419–3438 (2019).

16. Lemke, B. & Kjellstrom, T. Calculating Workplace WBGT from Meteorological Data: A Tool for Climate Change Assessment. *Industrial Health* **50**, 267–278 (2012).

17. Hallegatte, S. An Adaptive Regional Input-Output Model and its Application to the Assessment of the Economic Cost of Katrina. *Risk Analysis* **28**, 779–799 (2008).

18. Britz, W. & Roson, R. G-RDEM: A GTAP-Based Recursive Dynamic CGE Model for Long-Term Baseline Generation and Analysis. *Journal of Global Economic Analysis* **4**, 50–96 (2019).

19. Koks, E. E. *et al.* Regional disaster impact analysis: comparing input–output and computablegeneral equilibrium models. *Nat. Hazards Earth Syst. Sci.* **16**, 1911–1924 (2016).

20. Callahan, C. W. & Mankin, J. S. Globally unequal effect of extreme heat on economic growth. *Science Advances* **8**, eadd3726 (2022).

21. Hsiang, S. M. Temperatures and cyclones strongly associated with economic production in the Caribbean and Central America. *Proceedings of the National Academy of Sciences* **107**, 15367–15372 (2010).

22. Colacito, R., Hoffmann, B. & Phan, T. Temperature and Growth: A Panel Analysis of the United States. *Journal of Money, Credit and Banking* **51**, 313–368 (2019).

23. Inoue, H. & Todo, Y. Firm-level propagation of shocks through supply-chain networks. *Nat Sustain* **2**, 841–847 (2019).

24. Xie, W. *et al.* Decreases in global beer supply due to extreme drought and heat. *Nature Plants* **4**, 964–973 (2018).

25. Parsons, L. A., Shindell, D., Tigchelaar, M., Zhang, Y. & Spector, J. T. Increased labor losses and decreased adaptation potential in a warmer world. *Nat Commun* **12**, 7286 (2021).

26. Hsiang, S. *et al.* Estimating economic damage from climate change in the United States. *Science* **356**, 1362–1369 (2017).

27. Lenzen, M. Aggregating input–output systems with minimum error. *Economic Systems Research* **31**, 594–616 (2019).

28. Ara, K. The Aggregation Problem in Input-Output Analysis. *Econometrica* **27**, 257–262 (1959).

29. Fei, J. C.-H. A Fundamental Theorem for the Aggregation Problem of Input-Output Analysis. *Econometrica* **24**, 400–412 (1956).
